# Supplementary material for: Clinical and Laboratory Associations with Methotrexate Metabolism Gene Polymorphisms in Rheumatoid Arthritis
Source: J Pers Med. 2020 Sep 26;10(4):149. doi: 10.3390/jpm10040149 (PMC7712198; doi:10.3390/jpm10040149)
Supplement: Supplementary file 1 [file jpm-10-00149-s001.pdf]

Journal of Personalized Medicine

MDPI

Supplementary Material D'Cruz et al.

	rs4149081						
	GG (n=36)		GA (n=13)		AA (n=17)		ANOVA
	Mean	SD ±	Mean	SD ±	Mean	SD ±	p value
C-reactive protein (mg/L)	9.9	13.6	7.8	7.5	9.4	13.8	0.870
ESR (mm/h)	17.7	18.9	17.7	13.0	15.1	18.9	0.877
DAS-28 ESR	3.6	1.6	4.0	1.3	3.2	1.6	0.427
RBC (cells/mL)	4.4	0.4	4.3	0.4	11.5	29.8	0.245
Hb (g/100mL)	132.5	13.5	130.4	8.7	125.0	32.6	0.434
WBC (cells/mL)	22.5	90.6	7.4	1.3	6.3	2.9	0.644
Neutrophils (cells/mL)	4.7	1.7	4.9	1.0	4.1	2.2	0.344
Lymphocytes (cells/mL)	1.6	0.6	1.6	0.3	1.5	0.6	0.758
Platelets (cells/mL)	253.8	78.2	248.6	87.6	243.4	70.9	0.900
ALT (u/L)	23.0	12.6	18.8	6.8	24.3	12.6	0.425
AST (u/L)	24.1	9.3	23.1	7.9	25.9	11.4	0.717
ALP (u/L)	80.0	19.4	75.3	19.8	81.9	27.9	0.707
Bilirubin mg/L	6.7	2.6	8.1	6.2	11.9	10.1	**0.020**

	rs1476413						
	CC (n=8)		CT (n=16)		TT (n=42)		ANOVA
	Mean	SD ±	Mean	SD ±	Mean	SD ±	p value
C-reactive protein (mg/L)	5.2	8.8	10.5	13.4	9.7	12.8	0.603
ESR (mm/h)	8.9	7.5	18.3	15.5	18.1	19.5	0.382
DAS-28 ESR	2.3	1.5	4.0	1.6	3.6	1.5	**0.045**
RBC (cells/mL)	4.4	0.3	4.2	0.4	7.3	18.9	0.750
Hb (g/100mL)	138.0	14.1	127.5	10.3	129.6	22.8	0.450
WBC (cells/mL)	5.9	1.4	6.9	2.6	7.2	2.3	0.169
Neutrophils (cells/mL)	3.8	0.9	4.6	2.0	4.8	1.8	0.351
Lymphocytes (cells/mL)	1.5	0.5	1.6	0.4	1.6	0.6	0.850
Platelets (cells/mL)	230.2	105.1	253.2	81.6	252.7	71.2	0.744
ALT (u/L)	25.0	17.3	23.6	14.6	21.8	9.6	0.735
AST (u/L)	23.8	10.3	21.7	9.6	25.5	9.5	0.399
ALP (u/L)	75.1	21.0	77.8	17.9	81.0	23.4	0.733
Bilirubin mg/L	6.8	3.4	8.9	9.8	8.5	5.6	0.752

	rs2231142			
	GG (n=6)		GT (n=15)	
	Mean	SD ±	Mean	S[illegible]
C-reactive protein (mg/L)	3.6	3.8	4.8	[illegible]
ESR (mm/h)	11.5	9.7	10.3	[illegible]
DAS-28 ESR	4.0	1.9	3.3	[illegible]
RBC (cells/mL)	4.4	0.4	4.1	[illegible]
Hb (g/100mL)	136.5	8.6	127.3	[illegible]
WBC (cells/mL)	7.9	2.4	6.5	[illegible]
Neutrophils (cells/mL)	5.0	1.0	4.3	[illegible]
Lymphocytes (cells/mL)	2.1	1.2	1.4	[illegible]
Platelets (cells/mL)	272.5	67.0	230.1	[illegible]
ALT (u/L)	22.2	11.5	21.1	[illegible]
AST (u/L)	22.8	8.0	24.7	[illegible]
ALP (u/L)	78.3	26.5	66.6	[illegible]
Bilirubin mg/L	7.4	2.8	10.3	[illegible]

Supplementary Table S1. Methotrexate treated cohort SNP association with laboratory and clinical features. Descriptive statistics summary across n=66 methotrexate treated participants, showing specified polymorphism genotype group means and ANOVA analyses. Statistically significant ($p < 0.05$) associations highlighted in grey subsequently had p values adjusted for multiple testing (Table 3). ESR: erythrocyte sedimentation rate; DAS28: disease activity score across 28 joints; RBC, red blood cell (count); Hb: haemoglobin; WBC: white blood cells; ALT: alanine aminotransferase; AST: aspartate aminotransferase; ALP: alkaline phosphatase; SD: Standard deviation.

SNP	Metabolite (time pt.)	Units	Mean	±SD	Mean	±SD	Mean	±SD	ANOVA p value	Genotype Comparison	Bonferroni *p* value	Summary

**rs17421511**		GG (n=9)		GA (n=12)		AA (n=6)					
SPY (BL)	ug/L	980.9	415.1	1251	650.5	1706	472.2	0.059	GA vs. AA	0.326	ns
									GG vs. AA	0.056	ns
SPY (6wk)	ug/L	953.2	526.3	1144	768.1	2114	876.5	0.014	GA vs. AA	**0.013**	*
									GG vs. AA	0.145	ns

A

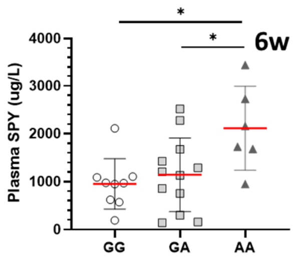

B

Supplementary Figure S1. Sulfasalzine treated cohort SNP associations. Plasma sulfapyridine (SPY) metabolite concentration which had statistically significant associations with the rs17421511 polymorphism in n=27 sulfasalazine treated participants. (A) Mean data for each genotype group with number of individuals per group (n=) indicated. Statistically significant differences between genotype group means were initially assessed by ANOVA and then adjusted by Bonferroni's multiple comparison test performed for specified genotype group mean comparisons; asterix used to summarise p values: (*) $p<0.05$. (B) 6 weeks follow up plasma concentration of sulfapyridine metabolite. Statistically significant differences between genotype group means are indicated by horizontal bars. Unlisted features had no statistically significant association with any SNP. Red horizontal bar represents genotype group mean; error bars represent standard deviation. BL: baseline; 6wk: six weeks follow up; SD: standard deviation; ns: not significant.

Time point	SNP	Mean	SD ±	Mean	SD ±	Mean	SD ±	ANOVA p value
	**rs4149081**	**GG (n=36)**		**GA (n=13)**		**AA (n=17)**		
Baseline		18.19	4.914	17.69	4.944	16.91	4.963	0.6772
6 week		18.02	5.877	16.25	3.773	18.08	3.252	0.5996
	**rs1476413**	**CC (n=8)**		**CT (n=16)**		**TT (n=42)**		
Baseline		18.44	4.419	18.91	4.18	17.2	5.215	0.4603
6 week		15	9.354	18.91	4.18	17.5	4.378	0.2932
	**rs2231142**	**GG (n=6)**		**GT (n=15)**		**TT (n=45)**		
Baseline		20.83	4.916	18.33	4.597	17.17	4.902	0.1993
6 week		21	5.477	18.33	3.427	17	5.209	0.2127
	**rs17421511**	**GG (n=26)**		**GA (n=31)**		**AA (n=9)**		
Baseline		17.21	4.708	18.39	4.897	17.22	5.652	0.6297
6 week		17.5	5.276	18.3	4.253	16.43	6.268	0.6727

Supplementary Table S2A. Methotrexate treated cohort prescription data. Mean methotrexate (mg/week) prescribing data per genotype group for the four polymorphisms rs4149081, rs1476413, rs2231142 and rs17421511, in n=66 methotrexate treated participants at baseline and 6 weeks follow up. SD: standard deviation.

		n prescribed MTX	median MTX dose/ mg per week (range)	n prescribed HCQ	median HCQ dose/ mg per day (range)	n prescribed LFD	median LFD dose/ ng per day (range)	n prescribed SFZ	median SFZ dose/ mg per day (range)
**Methotrexate prescribed subgroup A**	Baseline	66	20 (7.5-25)	26	200 (200-400)	-	-	11	1000 (500-1000)
(n=66)	6 weeks	51	20 (10-25)	20	200 (-)	-	-	9	1000 (500-1000)
**Sulfasalazine prescribed subgroup B**	Baseline	11	20 (15-25)	5	200 (-)	2	200 (-)	27	1000 (500-1000)
(n=27)	6 weeks	13	20 (10-25)	3	200 (-)	2	200 (-)	25	1000 (500-1000)

Supplementary Table S2B. Methotrexate and sulfasalazine treated subgroup co-prescription data. Median prescribing and co-prescribing data per study subgroup, in a subgroup of n=66 methotrexate and a subgroup of n=27 sulfasalazine treated participants at baseline and 6 weeks follow up.

Target SNP	Primer name	Primer sequence 5'-3'
rs246240	rs246240_FWD_M13	*CGCCAGGGTTTCCCAGTCACGAC*AAGCAACAGGGCAAACAAA
	rs246240_REV	TGCACCCAGCTGTTATCCT

	rs246240_SEQ	TGTCCAGCAGTAAGAGAT
rs717620	rs717620_F_M13	*CGCCAGGGTTTCCCAGTCACGAC*GCAGAACTTCTCCAGCATGAT
	rs717620_REV	AGGTCATCCTTTACGGAGAACAT
	rs717620_SEQ	TCATATTAATAGAAGAGTCT
rs3740065	rs3740065_F_M13	*CGCCAGGGTTTCCCAGTCACGAC*TTGTGTTGCTGCCACCTAGTG
	rs3740065_REV	GAGCTAGTTCCCTAGGATGGACAC
	rs3740065_SEQ	CCCTAGGATGGACACG
rs16853826	rs16853826_F_M13	*CGCCAGGGTTTCCCAGTCACGAC*GTCTAAAGTAGGCTGTGCTTGTTG
	rs16853826_REV	ACCCAATGCCTAGCACTCA
	rs16853826_SEQ	GAATGATTCCTGCAAA
rs4846051	rs4846051_F_M13	*CGCCAGGGTTTCCCAGTCACGAC*ATGAACCAGGGTCCCCACTC
	rs4846051_REV	GGGAGGAGCTGACCAGTGAAG
	rs4846051_SEQ	AAGAAAGTGTCTTTGAAGTC
rs17421511	rs17421511_F_M13	*CGCCAGGGTTTCCCAGTCACGAC*CGGTTCAGAGAGCTGGCTAGA
	rs17421511_REV	AGCATCCACTGCCCCTGT
	rs17421511_SEQ	TGCCCCTGTGTTTTC
rs1476413	rs1476413_F_M13	*CGCCAGGGTTTCCCAGTCACGAC*CCCCAGGGTTCCCACAGA
	rs1476413_REV	CTTAAGAGCAGGGCCCCAGTT
	rs1476413_SEQ	TGTTTGCTCAACGAAG
rs4149081	rs4149081_F_M13	*CGCCAGGGTTTCCCAGTCACGAC*TGCAGTATGGTATCAAGCAATCTC
	rs4149081_REV	CACATACAAATCCTAGCCATTTTC
	rs4149081_SEQ	TTCTATTATCTCTGATTTTT
rs10280623	rs10280623_F_M13	*CGCCAGGGTTTCCCAGTCACGAC*CCTCACAATCATGGCAGAAG
	rs10280623_REV	GGTCCTACAAACTGGTGATAACCT
	rs10280623_SEQ	TGACAGTCCACTGCATAA
rs2231142	rs2231142_F_M13	*CGCCAGGGTTTCCCAGTCACGAC*ATGTTGTGATGGGCACTCTGA

	rs2231142_REV	TTTTCCACATTACCTTGGAGTCTG
	rs2231142_SEQ	CGAAGAGCTGCTGAGAA
N/A	M13_BIOTIN	CGCCAGGGTTTTCCCAGTCACGAC

Supplementary Table S3. Pyrosequencing SNP primers. Primers used in pyrosequencing validation of SNP genotyping (M13 universal sequence shown in red/italics).

© 2020 by the authors. Submitted for possible open access publication under the terms and conditions of the Creative Commons Attribution (CC BY) license (http://creativecommons.org/licenses/by/4.0/).
